# Phylogenomic analysis, cryptic species discovery, and DNA barcoding of the genus *Cibotium* in China based on plastome data

**DOI:** 10.3389/fpls.2023.1183653

**Published:** 2023-06-06

**Authors:** Ri-Hong Jiang, Si-Qi Liang, Fei Wu, Li-Ming Tang, Bo Qin, Ying-Ying Chen, Yao-Heng Huang, Kai-Xiang Li, Xian-Chun Zhang

**Affiliations:** ^1^Guangxi Key Laboratory of Special Non-wood Forest Cultivation and Utilization, Guangxi Engineering and Technology Research Center for Woody Spices, Guangxi Forestry Research Institute, Nanning, China; ^2^State Key Laboratory of Systematic and Evolutionary Botany, Institute of Botany, The Chinese Academy of Sciences, Beijing, China; ^3^China National Botanical Garden, Beijing, China; ^4^College of Life Sciences, University of Chinese Academy of Sciences, Beijing, China; ^5^Beijing Botanical Garden, Beijing, China; ^6^Beijing Floriculture Engineering Technology Research Centre, Beijing, China; ^7^Guangxi Forestry Industry Group Stock Corporation, Nanning, China

**Keywords:** chloroplast genome, *Cibotium barometz*, *Cibotium sino-burmaense*, conservation, DNA barcoding, endangered species, germplasm resource, species diversity

## Abstract

Germplasm resources are the source of herbal medicine production. The cultivation of superior germplasm resources helps to resolve the conflict between long-term population persistence and growing market demand by consistently producing materials with high quality. The fern species *Cibotium barometz* is the original plant of cibotii rhizoma (“Gouji”), a traditional Chinese medicine used in the therapy of pain, weakness, and numbness in the lower extremities. Long-history medicinal use has caused serious wild population decline in China. Without sufficient understanding of the species and lineage diversity of *Cibotium*, it is difficult to propose a targeted conservation scheme at present, let alone select high-quality germplasm resources. In order to fill such a knowledge gap, this study sampled *C. barometz* and relative species throughout their distribution in China, performed genome skimming to obtain plastome data, and conducted phylogenomic analyses. We constructed a well-supported plastome phylogeny of Chinese *Cibotium*, which showed that three species with significant genetic differences are distributed in China, namely *C. barometz*, *C. cumingii*, and *C. sino-burmaense* sp. nov., a cryptic species endemic to NW Yunnan and adjacent regions of NE Myanmar. Moreover, our results revealed two differentiated lineages of *C. barometz* distributed on the east and west sides of a classic phylogeographic boundary that was probably shaped by monsoons and landforms. We also evaluated the resolution of nine traditional barcode loci and designed five new DNA barcodes based on the plastome sequence that can distinguish all these species and lineages of Chinese *Cibotium* accurately. These novel findings on a genetic basis will guide conservation planners and medicinal plant breeders to build systematic conservation plans and exploit the germplasm resources of *Cibotium* in China.

## Introduction

Traditional Chinese herbal medicine plays an indispensable role in the treatment of multiple diseases in China and other developing countries (Newman et al., 2008). Apart from the traditional usage, many medicinal plants, such as *Artemisia annua* L. (artemisinin, Tu, 2016), *Huperzia javanica* (Sw.) C. Y. Yang (Huperzine A, Zangara, 2003; it was mistakenly named as *Huperzia serrata* (Thunb.) Trevis in many studies, Chen et al., 2021), and *Panax notoginseng* (Burk.) F. H. Chen (Notoginseng triterpenes, Huang et al., 2021), are also the source of modern pharmaceuticals generating increasing attention. Although China harbors abundant medicinal plant diversity, the original species of many commonly used herbal medicines are facing the risk of population decline and even extinction under growing demand (Chen et al., 2016). Germplasm resources are a major determinate of medicine production (Ma and Xiao, 1998; Huang et al., 2008; Zhang and Jiang, 2021; Meng et al., 2023). Cultivation of specific high-quality germplasm resources will not only resolve the present conflict between conservation and exploitation but also ensure a steady production of high-quality medicines (Ma and Xiao, 1998; Chen et al., 2016). Therefore, clarifying genetic background and diversity is the basic and crucial step in achieving sustainable utilization of medicinal plants and provides implications for the collection, identification, evaluation, and conservation of germplasm resources (Schoen and Brown, 1993; Ma and Xiao, 1998; Yu et al., 2013; Khoury et al., 2022).

**Figure 1:**
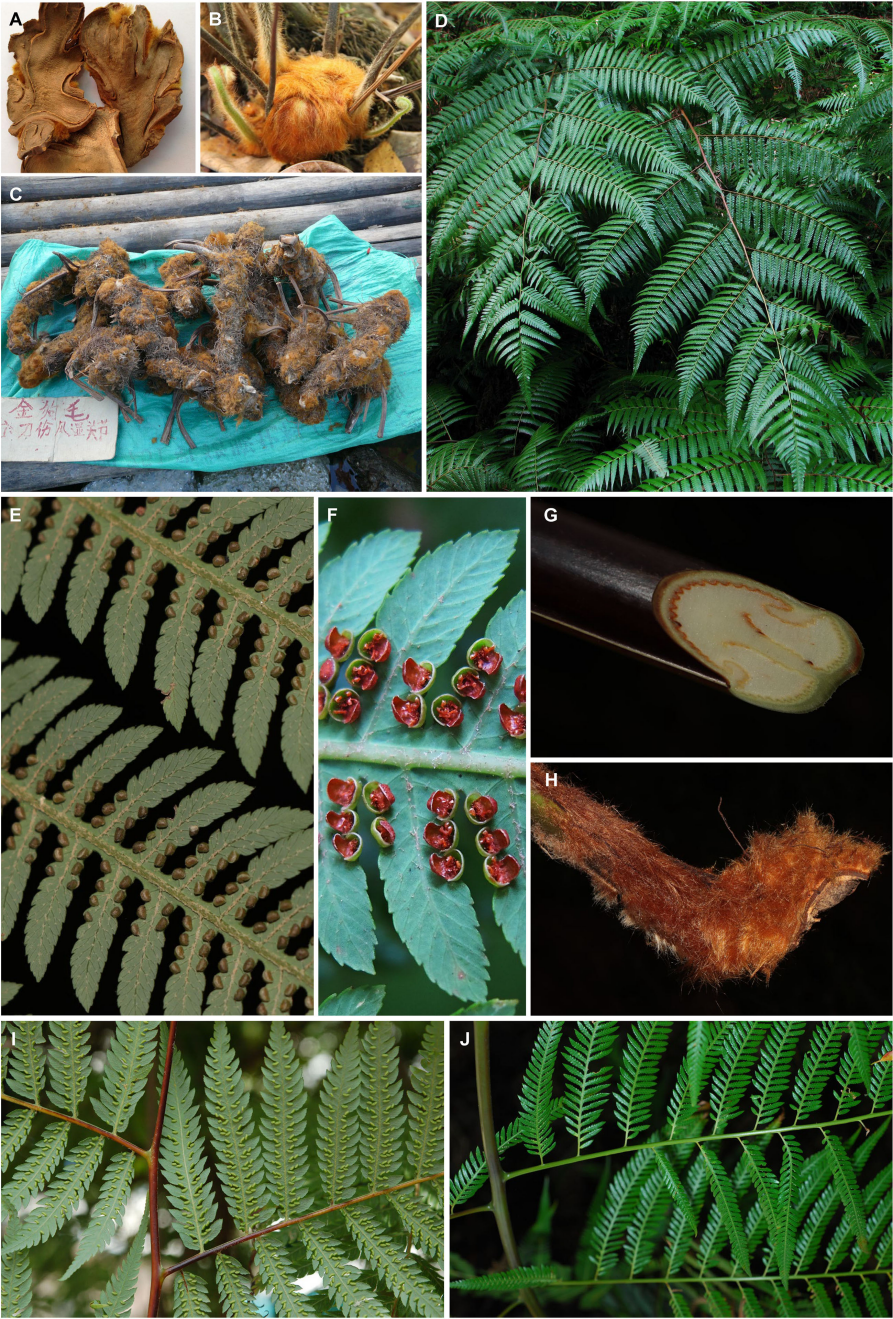
Morphology of *Cibotium* plants from China. **(A)** Dry sliced rhizomes of *C. barometz*, “Gouji.” **(B)** Rhizome, stipes, and young fronds of *C. barometz* covered with golden filiform hairs. **(C)** Rhizomes of *C. barometz* sold as medicinal herbs at a village fair. **(D)** Fronds of *C. barometz*. **(E)** Veins, hairs, and unopened sori on the abaxial surface of the pinnae of *C. barometz*. **(F)** Opened sori of *C. barometz*. **(G, H)** Cross section and basal part of the stipe of *C. barometz*. **(I, J)** Basal part of pinna in *C. barometz* and *C. cumingii* showing the difference of basal pinnules on the basiscopic side. Photographs by R.-H. Jiang **(A, B, D, F),** X.-C. Zhang **(C, I, J)**, and Q.-K. Ding **(E, G, H)**.

Cibotium barometz (L.) J. Sm. is the original species (**Figures 1A–I**) of traditional medicine, cibotii rhizoma (“Gouji”, **Figure 1A**), the processed rhizome of which can be used in the therapy of pain, weakness, and numbness of the lower extremities (Chinese Pharmacopoeia Commission, 2020). Phytochemical research has shown that the extract of its rhizomes is rich in active compounds such as pterosins, terpenes, steroids, flavonoids, glucosides, phenolic acids, and pyrones (Xu et al., 2012). Bioactivity experiments supported the efficacy, including the treatment of osteoporosis and osteoarthritis, antioxidant and antimicrobial activities, as well as abirritation (Ju et al., 2005; Cuong et al., 2009; Zhao et al., 2011; Li et al., 2014; Fu et al., 2017; Heng et al., 2020; Sun, 2021). Pot cultures and crafts of this species are also popular on the market because of their large, elegant evergreen fronds and stump-like rhizomes covered with long, soft, golden hairs resembling gold-hair dogs (**Figures 1B–D**). Medicinal and ornamental values have resulted in the destructive plunder of abundant natural resources by *C. barometz* in China. Investigation has shown that uncontrolled collection and habitat deconstruction are major threats to its population survival (Zhang et al., 2002).

*C. barometz* is listed in Appendix II of CITES (Zhang et al., 2002; https://cites.org/eng/app/appendices.php), and the genus *Cibotium* is listed in the Grade II Category of the List of National Key Protected Wild Plants of China (State Forestry and Grassland Administration and the Ministry of Agriculture and Rural Affairs, P. R. China, 2021). Although the Chinese government has attached great importance to this genus, researchers are unable to specify which species or populations are key units awaiting conservation grounded in present knowledge. Such a phenomenon could lead to the waste of protective efforts and affect the maximization of medical value. Previous studies have shown that the genus *Cibotium* (Cibotiaceae, a member of the tree fern clade) comprises ca. 9–12 species distributed in tropical and subtropical regions of Asia, Central America, and the Hawaiian Islands (Holttum, 1963; Palmer, 1994; Korall et al., 2006; Smith et al., 2006; Geiger et al., 2013), three Asian members of which form a monophyletic clade (Geiger et al., 2013). Two species, *C. barometz* and *C. cumingii* Kunze, were recognized from China (Zhang and Nishida, 2013). The former is widespread in southern China and extends to northeastern India and Malaysia, while the latter is only known from Taiwan Island in China, as well as the Ryukyu and Philippine Islands (Holttum, 1963; Zhang and Nishida, 2013). However, the geographical pattern of genetic diversity and differentiation of *C. barometz* has not been explored throughout its wide distribution, let alone talking about specific populations awaiting conservation and the variation of medicinal values among different regions accurately. In addition, though individuals can grow up to three meters tall, merely a handful of morphological traits can be applied to the discrimination of *C. barometz* and *C. cumingii* (Holttum, 1963; Zhang and Nishida, 2013). Therefore, whether recognized phenotypes could adequately reflect the diversity of this genus also remains to be verified.

In previous studies, several chloroplast DNA (cpDNA) fragments have been applied to the phylogenetic construction of the tree fern clade, including *Cibotium* (Korall et al., 2006; Geiger et al., 2013). However, the informative variation sites provided by these loci are insufficient to illuminate the relationship within Chinese *Cibotium*. With advantages including low requirements for material quality, low costs, and rich variable sites, the chloroplast genome (plastome) has been widely utilized for phylogenetic reconstruction at different levels as well as species delimitation of closely related species in recent years (e.g., Hammer et al., 2019; Wei and Zhang, 2020; Ji et al., 2021; Du et al., 2022; Xi et al., 2022; Zhang et al., 2022; Yang et al., 2023). Therefore, analyzing genetic variations of plastomes obtained from multiple samples representing the major distribution will help us clarify species diversity and the phylogeographical pattern of Chinese *Cibotium*, which are crucial to suggesting reasonable conservation units and germplasm resources suitable for sustainable medicine production. Furthermore, plastome data can not only be applied to develop traditional DNA barcodes but also used as a single genetic marker, namely ultra-barcodes (Nock et al., 2011; Kress et al., 2015; Hollingsworth et al., 2016), which largely benefits the identification and evaluation of medical plant products, especially partial organs and tissues lacking diagnostic phenotypic features (Park et al., 2021; Qin et al., 2022; Wang et al., 2022; Wei et al., 2022).

In this study, we performed genome skimming and assembled complete plastomes of representative samples of *C. barometz* and relatives throughout the distribution range in China and adjacent areas. We aimed to 1) compare structure and composition variations of plastomes among Chinese *Cibotium* species; 2) propose a phylogeny-based species delimitation; 3) investigate the geographical pattern of variation of *C. barometz* based on plastome data; and 4) suggest candidate barcodes for specific species and lineage identification of Chinese *Cibotium*. We believed that our findings would benefit the conservation and breeding of this important medicinal plant taxon and provide insights into the systematics and evolution of ferns.

## Materials and methods

### Taxon sampling, DNA extraction, and Illumina sequencing

**Figure 2:**
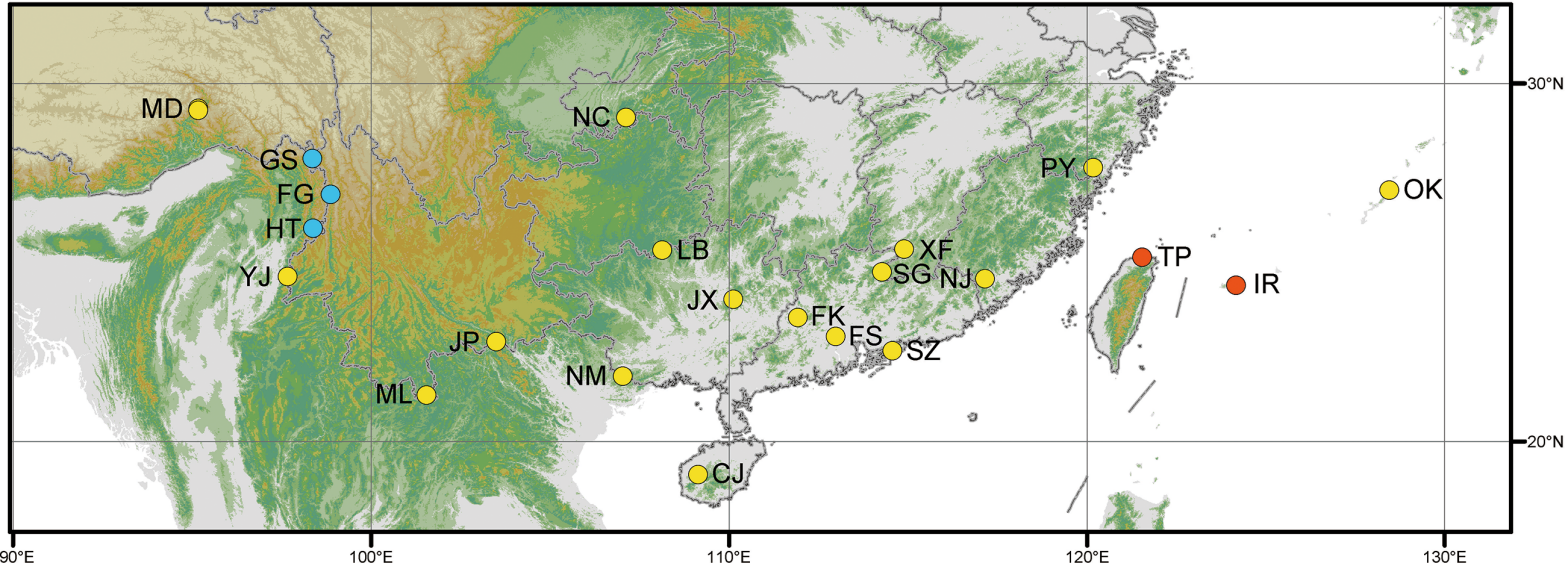
Map showing the distribution of *Cibotium* samples in this study. Yellow, blue, and red dots represent localities of *C. barometz*, *C. sino-burmaense*, and *C. cumingii*, respectively. The code of each sampling locality means the same as in **Table 1**.

Frond tissues of 25 *Cibotium* individuals were collected for genome skimming sequencing throughout the distribution range of China and adjacent regions (**Table 1**; **Figure 2**). Most accessions were fresh fronds dried using silica gel and preserved at 4 °C, except for five samples obtained from specimens deposited in the herbarium PE (**Table 1**). Based on the presence or lack of basal pinnules on the basiscopic side of pinnae on voucher specimens (**Figures 1I, J**, Zhang and Nishida, 2013), 25 samples were sorted into 23 individuals of *C. barometz* and two individuals of *C. cumingii* preliminarily.

**Table 1:** Summary of sampling information and plastome characteristics in this study.

Species	Code	Locality	Voucher	Total genome size (bp)	GC content (%)	LSC size (bp)	SSC size (bp)	IR size (bp)	Pseudo gene
*C. sino-burmaense*	FG1	Fugong, Yunnan, China	12831-1, X. C. Zhang	162,115	41.4	85,637	22,064	27,207	*matK*
*C. sino-burmaense*	FG2	Fugong, Yunnan, China	12831-2, X. C. Zhang	162,108	41.4	85,634	22,062	27,206	*matK*
*C. sino-burmaense*	GS1	Gongshan, Yunnan, China	12880-1, X. C. Zhang	162,116	41.4	85,636	22,066	27,207	*matK*
*C. sino-burmaense*	GS2	Gongshan, Yunnan, China	12880-2, X. C. Zhang	162,112	41.4	85,634	22,064	27,207	*matK*
*C. sino-burmaense*	HT	Htawgaw, Kachin, Myanmar	26496, G. Forrest*	162,206	41.4	85,645	22,056	27,248	*matK*
*C. barometz*	JP1	Jinping, Yunnan, China	PT388-1, Z. Y. Li	165,683	41.7	85,722	22,059	28,951	*matK*
*C. barometz*	ML	Mengla, Yunnan, China	5640, Y. Shang	165,665	41.7	85,675	22,064	28,963	*matK*
*C. barometz*	YJ	Yingjiang, Yunnan, China	7947, X. C. Zhang & Z. Y. Guo	166,087	41.7	85,670	22,063	29,177	*matK*
*C. barometz*	MD1	Medog, Xizang, China	05237, B. S. Li & S. Z. Cheng*	166,019	41.7	85,669	22,066	29,142	*matK*
*C. barometz*	MD2	Medog, Xizang, China	13841-6, X. C. Zhang & al.	166,099	41.7	85,683	22,068	29,174	*matK*
*C. barometz*	NM	Ningming, Guangxi, China	7897, X. C. Zhang & al.	165,767	41.7	85,673	22,066	29,014	*matK*
*C. barometz*	JX	Jinxiu, Guangxi, China	6042, X. C. Zhang	166,054	41.7	85,674	22,066	29,157	*matK*
*C. barometz*	NC	Nanchuan, Chongqing, China	22, Z. Y. Liu	165,653	41.7	85,694	22,017	28,944	*matK*
*C. barometz*	PY	Pingyang, Zhejiang, China	s.n.-2, H. Zhang & J. C. Zhang	166,033	41.7	85,667	22,054	29,156	
*C. barometz*	XF	Xinfeng, Jiangxi, China	lxp-13-22042, Ecology Internship Group, SYSU	166,051	41.7	85,673	22,054	29,162	*matK*
*C. barometz*	SZ	Shenzhen, Guangdong, China	6571, R. H. Jiang & al.	165,983	41.7	85,656	22,053	29,137	*matK*
*C. barometz*	FS	Foshan, Guangdong, China	5493, X. C. Zhang & al.	165,669	41.7	85,686	22,053	28,965	
*C. barometz*	FK	Fengkai, Guangdong, China	5454, X. C. Zhang & al.	166,050	41.7	85,672	22,054	29,162	*matK*
*C. barometz*	SG	Shaoguan, Guangdong, China	CBL006, X. C. Zhang & al.	166,194	41.7	85,649	22,054	29,219	*matK*
*C. barometz*	LB	Libo, Guizhou, China	11209, X. C. Zhang & al.	166,016	41.7	85,781	22,054	29,142	*matK*
*C. barometz*	NJ	Nanjing, Fujian, China	SH2015120802, X. P. Wei	166,030	41.7	85,664	22,054	29,156	
*C. barometz*	CJ	Changjiang, Hainan, China	1558, X. C. Zhang & al.	166,443	41.7	85,697	22,048	29,349	
*C. barometz*	OK	Okinoerabu Island, Japan	2410, Y. Saiki*	166,041	41.6	85,673	22,052	29,158	*matK*
*C. cumingii*	TP	Taipei, Taiwan, China	1113, W. C. Leong*	165,077	41.7	85,648	22,067	28,681	*matK*
*C. cumingii*	IR	Iriomote Island, Japan	s.n., Y. Saiki*	165,221	41.7	85,641	22,062	28,759	*matK*

An asterisk (*) after voucher information indicates that the tissue for Illumina sequencing was obtained from specimen deposited in herbarium.

All the tissue samples were sequenced at the Novogene Corporation (Beijing, China). Total genomic DNA was extracted with a modified CTAB procedure (Doyle and Doyle, 1987). Libraries with an insert size of 350 bp were constructed using a TruSeq Nano DNA HT Sample Preparation Kit (Illumina, San Diego, California, USA) following the manufacturer’s recommendations. Paired-end reads (PE150) were then sequenced on an Illumina NovaSeq 6000 platform. After quality control of raw reads using ng_QC v.2.0 developed by Novogene Corporation with the default settings, we obtained ca. 2 to 4 Gb of clean reads for each sample.

### Plastomes assembly and annotation

We *de novo* assembled plastomes of all our samples with clean reads using the GetOrganelle toolkit (Jin et al., 2020) with recommended parameters. The complete plastome of *C. barometz* (NC_037893, Liu et al., 2018) downloaded from the GenBank database was used as a reference during assembly and annotation. Assembly errors were identified in the initial assembly contigs and manually corrected by mapping raw reads to assembled sequences with Geneious v.11.1.4 (Kearse et al., 2012). Boundaries of large single-copy (LSC), small single-copy (SSC), and two inverse repeat regions (IRs) were detected using RepeatFinder v.1.0.1 (Volfovsky et al., 2001). Genome annotation was performed with GeSeq (Tillich et al., 2017) and Geneious v.11.1.4 (Kearse et al., 2012). Protein-coding sequences were checked against the National Center for Biotechnology Information (NCBI) database and manually corrected. tRNAs were confirmed with tRNAscan-SE v2.0.3 (Lowe and Chan, 2016). The final circular map of the plastome was visualized using OGDraw v.1.3.1 (Greiner et al., 2019). We also used the program LAGAN (Brudno et al., 2003) in mVISTA to compare the gene order and structure among different species with the plastome sequence alignment generated by MAFFT v.7.313 (Katoh and Standley, 2013).

### Phylogenetic analyses

The full-length plastome sequences of all *Cibotium* samples and the reference (NC_037893), as well as three outgroup species from the tree fern clade, i.e., *Alsophila spinulosa* (NC_012818), *Sphaeropteris brunoniana* (NC_051561), and *Plagiogyris euphlebia* (NC_046784), were aligned with MAFFT v.7.313 (Katoh and Standley, 2013) after the removal of one IR region. According to the phylogeny of Korall et al. (2006), *Alsophila*, *Sphaeropteris*, and *Cibotium* all belong to the “core” tree fern clade, while *Plagiogyris* is more distantly related to the three genera. The alignment was then filtered using GBLOCKS v.0.91b (Castresana, 2000) to remove ambiguously aligned regions. We also extracted the protein-coding genes of each plastome with a python script (https://github.com/Kinggerm/PersonalUtilities/blob/master/get_annotated_regions_from_gb.py) and concatenated all these single gene alignments to build a protein-coding gene dataset for phylogenetic analyses. The best-fitting nucleotide substitution models of the full-length and protein-coding gene alignments were determined as TVM + F + G4 and GTR + F + G4, respectively, based on the Bayesian information criterion (BIC) by ModelFinder (Kalyaanamoorthy et al., 2017). Maximum likelihood (ML) analysis was performed with both datasets using IQ-TREE v.1.6.8 (Nguyen et al., 2015) with 10,000 ultrafast bootstrap replicates (Minh et al., 2013). Bayesian inference (BI) analysis was performed with the protein-coding gene dataset using MrBayes v.3.2.6 (Ronquist et al., 2012). One cold and three hot chains were run for 2,000,000 generations, with sampling taken every 1,000 generations and a burn-in of 25%. The convergence of Markov chain Monte Carlo runs was checked with Tracer v.1.7.1 (Rambaut et al., 2018) to ensure that the effective sampling size (ESS) of all parameters was above 200. Phylogenetic trees were all visualized, rooted with *P. euphlebia*, and edited in FigTree v.1.4.2 (Rambaut, 2014).

### Candidate barcoding region detection and verification

To identify candidate regions for species and even lineage discrimination in Chinese *Cibotium* plants, we first used DnaSP v.6.12.03 (Rozas et al., 2017) to evaluate π of the plastome sequence alignment of *C. barometz* with a window length of 800 bp and a step size of 200 bp. Nucleotide polymorphism sites fixed to specific species and lineages were also identified by checking the alignment, including all *Cibotium* samples. Additionally, the feasibility and convenience of PCR amplification in practice were also taken into consideration; therefore, the chosen barcode regions are all shorter than 800 bp in length and have conservative flanks suitable for primers to combine with. Candidate loci meeting all these requirements were finally selected, PCR primers of which were designed using Primer3 v.2.3.7 (Koressaar and Remm, 2007; Untergasser et al., 2012).

We extracted sequences of newly selected loci and nine cpDNA markers (*atpA*, *atpB*, *rbcL*, *rps4*, *rbcL-accD*, *rbcL-atpB*, *trnG-trnR*, *trnL-trnF*, and *rps4-trnS*) applied in previous studies (Korall et al., 2006; Geiger et al., 2013) from all our samples, including other accessible data of *C. barometz* and *C. cumingii* on GenBank and aligned them. We counted the number of variable sites with MEGA v.10.1.6 (Kumar et al., 2018) and performed ML analysis on each alignment of the 14 barcode loci and some of their combinations, including outgroups, following the same procedure as mentioned above. We compared the topologies of the resulting phylogenetic trees to the ones built with the plastome dataset to evaluate the effectiveness of these loci in species and lineage discrimination. Multiple individuals of a specific taxon resolved as monophyletic with bootstrap support over 50% and were treated as successfully discriminated.

## Results

### Plastome characteristics of *Cibotium*

**Figure 3:**
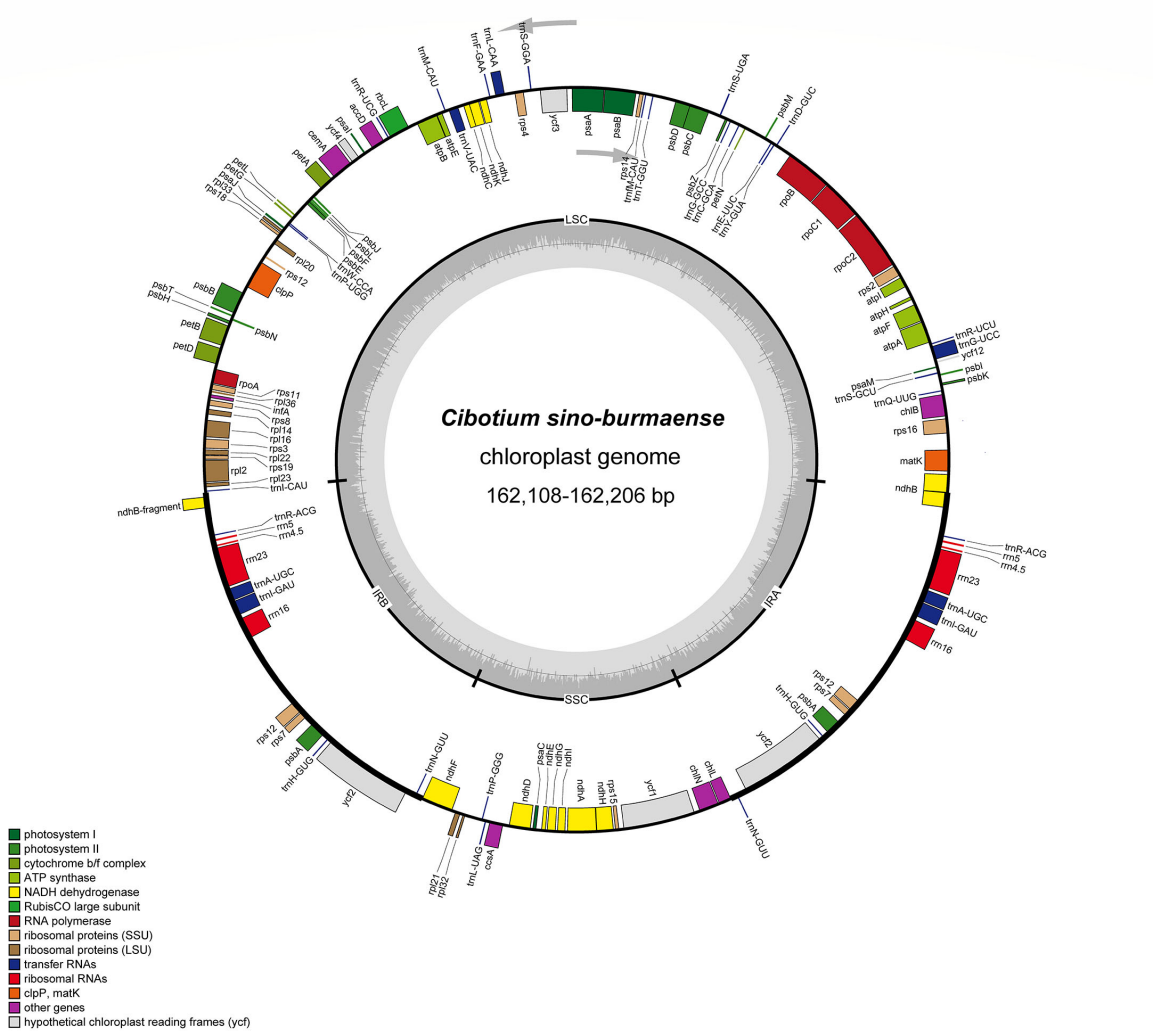
Plastome map of *Cibotium sino-burmaense*. Arrows indicate the direction of gene transcription. The dark gray area of the inner circle shows GC content variation among different regions of the plastome.

**Figure 4:**
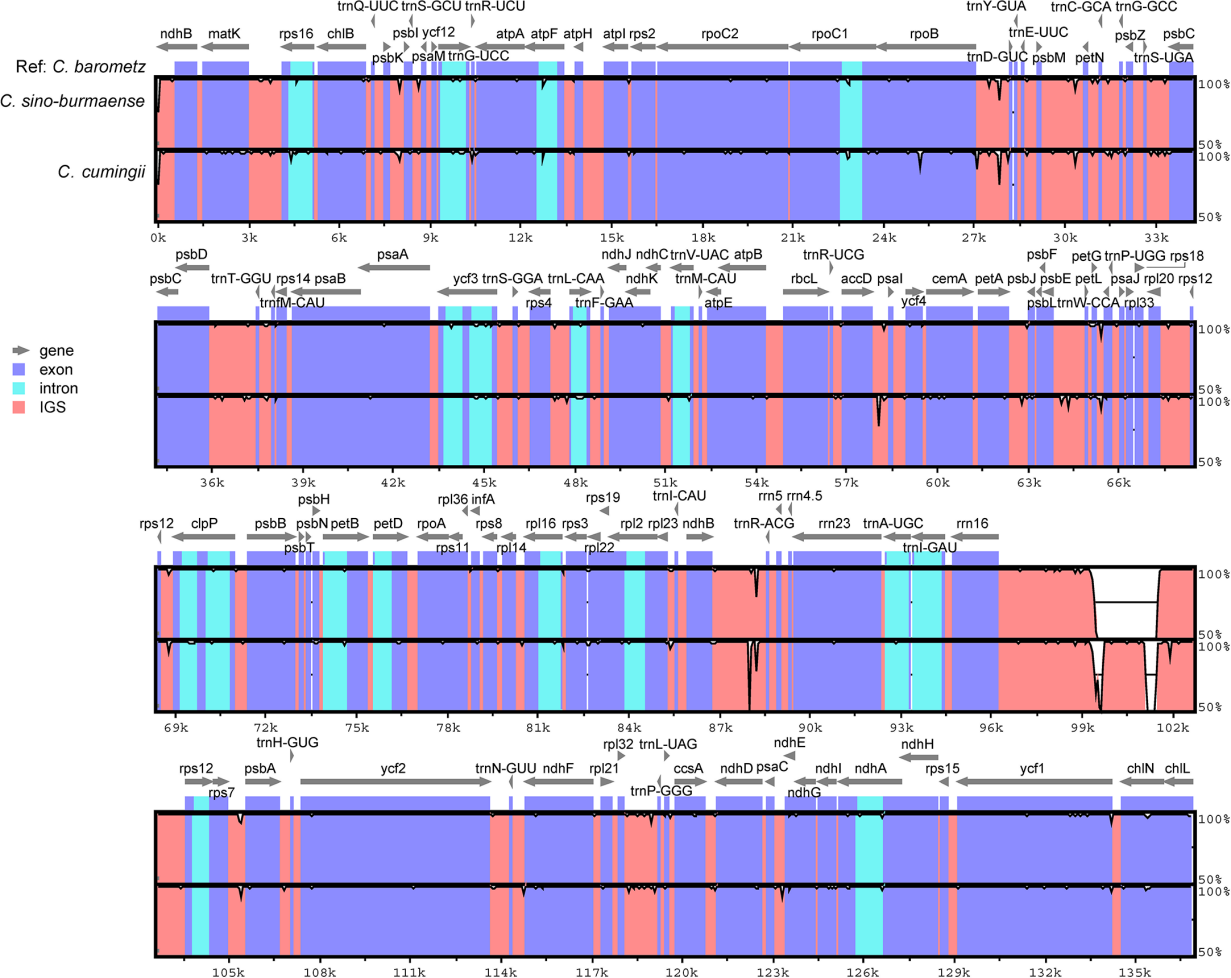
Sequence identity plot comparing the plastome sequence and constitution of three Chinese *Cibotium* species with *C. barometz* as a reference. Each sequence starts with the beginning of LSC and ends at the end of SSC. Gray arrows indicate genes by their orientation. A cut-off of 50% identity was used for the plot, and the Y-axis represents the percent identity ranging from 50% to 100%.

Complete chloroplast genomes of 25 sampled individuals of *Cibotium* were obtained and assembled into circular molecules comprising one LSC, one SSC, and two IRs (**Figure 3**; **Table 1**), which are all typical quadripartite structures. Complete plastomes of *C. cumingii* and the majority of *C. barometz* ranged from 165,077 to 166,443 bp in length with very similar GC contents of ca. 41.7%, except for five “*C. barometz*” samples probably of an unknown species collected from NW Yunnan and NE Myanmar with significantly shorter length (162,108–162,206 bp) and lower GC content (41.4%). The length of LSC (85,634–85,781 bp) and SSC (22,017–22,067 bp) is rather stable among all accessions, whereas IR size varies among samples of *C. cumingii* (28,681–28,759 bp), most *C. barometz* (28,944–29,349 bp), and those Yunnan–Myanmar samples (27,206–27,248 bp) with clearly no difference. The boundaries of IRs are the same among all samples, without any expansion or contraction. In comparison, the intergeneric region between *rrn16* and *rps12* varies greatly among species (**Figure 4**), which mainly results in IR size variation.

All the plastomes encode a total of 117 unique genes in identical order, including 85 protein-coding genes, 28 tRNA genes, and four rRNA genes (**Table 2**; **Figure 3**), which are generally consistent with the reference (Liu et al., 2018). In most samples of *C. barometz*, the annotated *matK* gene region could not be successfully translated into protein (pseudogenization) because of an early termination resulting from the missing one or two nucleotides (**Table 1**). The gene *ycf2*, which was predicted as a pseudogene (6,250 bp) due to a codon shift mutation in the reference plastome of *C. barometz* (NC_037893, Liu et al., 2018), is normal (6,249 bp) in all the samples of this study. All four rRNA genes, five tRNA genes (t*rnA-UGC*, *trnH-GUG*, *trnI-GAU*, *trnN-GUU*, and *trnR-ACG*), and three protein-coding genes (*rps7*, *psbA*, and *ycf2*) are totally duplicated, whereas *ndhB* and *rps12* have only one incomplete duplication.

**Table 2:** Genes in the plastome of *Cibotium* plants from China.

Function	Group of genes	Gene names
Protein synthesis and DNA replication	Ribosomal RNAs	*rrn4.5* (×2), *rrn5* (×2), *rrn16* (×2), *rrn23* (×2)
	Transfer RNAs	*trnA-UGC* ^a^ (×2), *trnC-GCA*, *trnD-GUC*, *trnE-UUC*, *trnF-GAA*, *trnfM-CAU*, *trnG-GCC*, *trnG-UCC* ^a^, *trnH-GUG* (×2), *trnI-CAU*, *trnI-GAU* ^a^ (×2), *trnL-CAA* ^a^, *trnL-UAG*, *trnM-CAU*, *trnN-GUU* (×2), *trnP-GGG*, *trnP-UGG*, *trnQ-UUG*, *trnR-ACG* (×2), *trnR-UCG*, *trnR-UCU*, *trnS-GCU*, *trnS-GGA*, *trnS-UGA*, *trnT-GGU*, *trnV-UAC* ^a^, *trnW-CCA*, *trnY-GUA*
	Large subunit of ribosome	*rpl2* ^a^, *rpl14*, *rpl16* ^a^, *rpl20*, *rpl21*, *rpl22*, *rpl23*, *rpl32*, *rpl3*3, *rpl36*
	Small subunit of ribosome	*rps2*, *rps3*, *rps4*, *rps7* (×2), *rps8*, *rps11*, *rps12*^a,c^, *rps14*, *rps15*, *rps16* ^a^, *rps18*, *rps19*
	RNA polymerase	*rpoA*, *rpoB*, *rpoC1* ^a^, *rpoC2*
Photosynthesis	Photosystem I	*psaA*, *psaB*, *psaC*, *psaI*, *psaJ*, *psaM*
	Photosystem II	*psbA* (×2), *psbB*, *psbC*, *psbD*, *psbE*, *psbF*, *psbH*, *psbI*, *psbJ*, *psbK*, *psbL*, *psbM*, *psbN*, *psbT*, *psbZ*
	NADH-dehydrogenase	*ndhA* ^a^, *ndhB* ^a^, *ndhC*, *ndhD*, *ndhE*, *ndhF*, *ndhG*, *ndhH*, *ndhI*, *ndhJ*, *ndhK*
	Cytochrome b6/f complex	*petA*, *petB* ^a^, *petD* ^a^, *petG*, *petL*, *petN*
	ATP synthase	*atpA*, *atpB*, *atpE*, *atpF* ^a^, *atpH*, *atpI*
	Large subunit of rubisco	*rbcL*
Miscellaneous function	Translation initiation factor	*infA*
	Acetyl-CoA carboxylase	*accD*
	Cytochrome c biogenesis	*ccsA*
	Maturase	*matK*
	ATP-dependent protease	*clpP* ^b^
	Envelope membrane protein	*cemA*
	Photochlorophyllide reductase	*chlB*, *chlL*, *chlN*
Unknown function	Conserved hypothetical open reading frames	*ycf1*, *ycf2* (×2), *ycf3* ^b^, *ycf4*, *ycf12*

a Gene containing one intron.

b Gene containing two introns.

c Trans-spliced gene.

### Phylogenomic relationship within *Cibotium*

**Figure 5:**
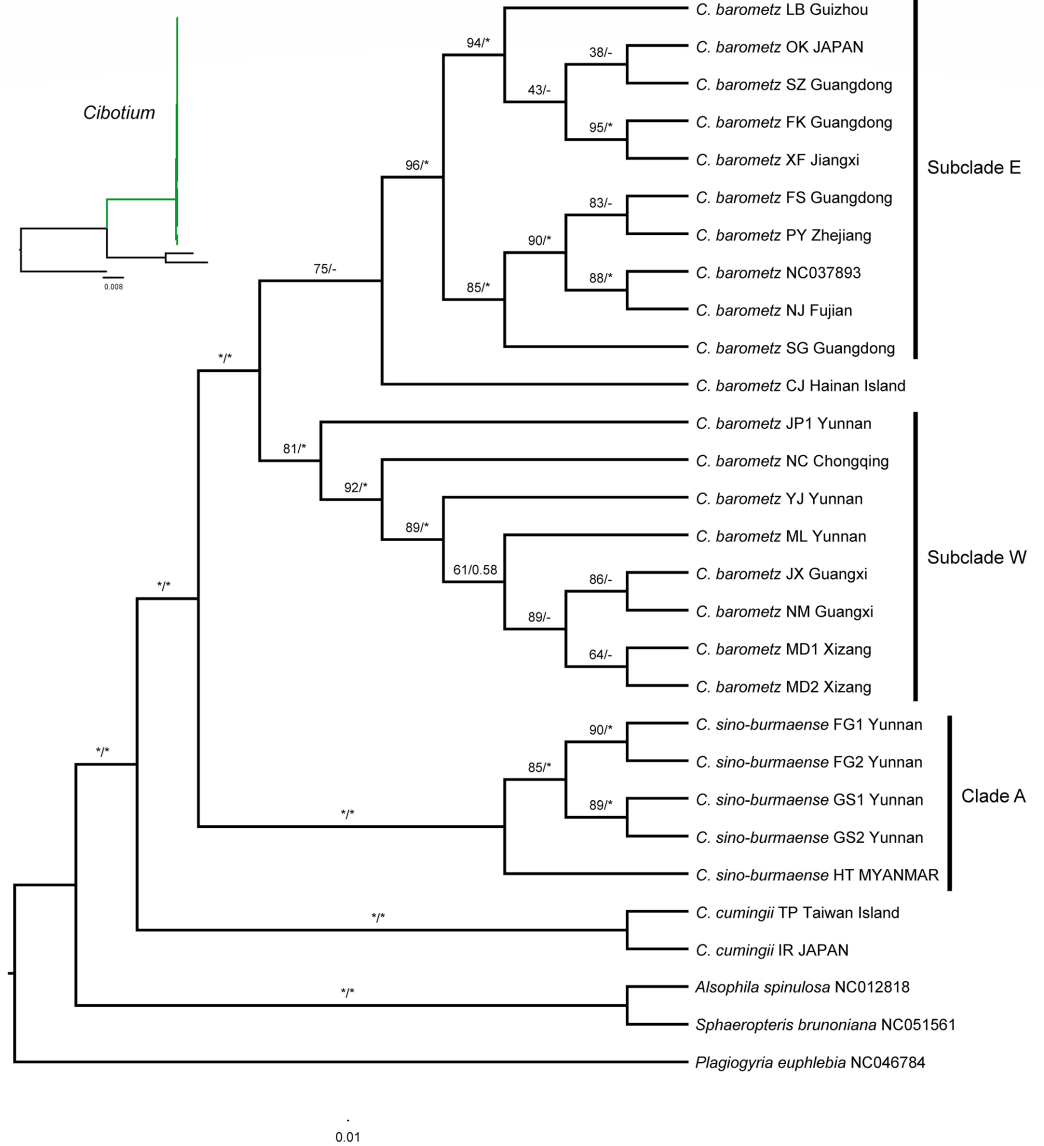
Maximum likelihood cladogram of the *Cibotium* plant from China and adjacent regions inferred from 85 concatenated protein-coding genes. The numbers above branches are bootstrap values (MLBS) and posterior probabilities (BIPP). An asterisk (*) indicates MLBS = 100% or BIPP = 1.0. En-dash (–) indicates the lack of support value. The corresponding phylogram showing branch length is placed in the upper left corner.

Phylogenetic trees (**Figures 5**; **S1**) built with ML and BI analyses based on both full-length (136,298 bp) and protein-coding genes (73,080 bp) datasets showed generally similar topologies and strongly supported the monophyly of *C. cumingii*, most *C. barometz*, as well as the five “*C. barometz*” samples from NW Yunnan and NE Myanmar. The clade formed by the five Yunnan–Myanmar samples (Clade A, **Figure 5**) was sister to the clade including all the other accessions of *C. barometz.* The remaining samples of *C. barometz* except the one collected on Hainan Island could be further divided into two lineages, i.e., Subclade E, including samples from SE China (Zhejiang, Jiangxi, Fujian, Guangdong, and Guizhou) and the Ryukyu Islands, and Subclade W, including samples from SW China (Chongqing, Guangxi, Yunnan, and Xizang). The Hainan sample clustered within Subclade E based on the protein-coding gene dataset with low support value (**Figure 5**) but became a sister to the combination of Subclade E and Subclade W (MLBS = 26) based on the full-length dataset (**Figure S1**). Therefore, although the relationship was not consistently resolved, both results reflected the divergence among the southeastern and southwestern subclades as well as the Hainan sample within *C. barometz*.

### DNA barcodes for *Cibotium* species discrimination

Based on phylogenetic results (**Figures S2A–I**), only four (*trnL-trnF*, *trnG-trnR*, *rps4-trnS*, and *rbcL-accD*) of the nine traditional cpDNA loci are effective in the identification of *C. cumingii*, while only the first two of the four could further discriminate Yunnan–Myanmar *Cibotium* correctly. None of them accurately depicted the intraspecies divergence within *C. barometz*. We also tested the concatenated dataset of *trnL-trnF*, *trnG-trnR*, *rps4-trnS*, and *rbcL-accD* and found poor resolution among all *C. barometz*, including those Yunnan–Myanmar samples (**Figure S2J**).

**Figure 6:**
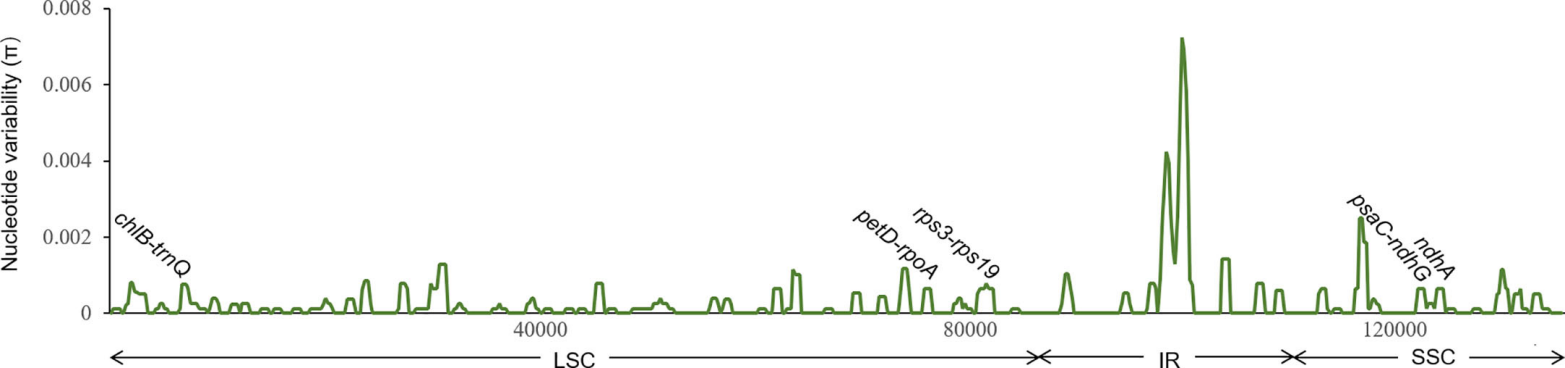
Sliding window analysis of 19 plastomes of *Cibotium barometz* (window length: 800 bp, step size: 200 bp). X and Y axes indicate the position of the midpoint of a window and nucleotide variability (π) of each window, respectively. Those marked fragments show the position of five newly designed DNA barcodes for inter- and intra-species discrimination in Chinese *Cibotium*.

The nucleotide variability of *C. barometz* plastomes is shown in **Figure 6**. Variable regions were distributed evenly along the plastome with π value less than 0.002 except for a highly variable region within IR between *rrn16* and *rps12*. Five fragments (**Figure 6**) with moderate variation for species and lineage discrimination as well as suitable length and flanks for PCR amplification were chosen as candidate DNA barcode loci. Comparing with the nine old cpDNA loci, these new barcodes showed higher variability among the Chinese *Cibotium* species (**Table 3**) and were all capable of correctly assigning individuals of *C. cumingii*, Yunnan–Myanmar *Cibotium*, and two different lineages within *C. barometz* into respective clades (**Figures S2K–O**). The performances of *rps3-rps19* and *psaC-ndhG* are the best among the five because each taxon was resolved as monophyletic with bootstrap support over 50%. The Hainan sample with an uncertain phylogenetic position was clustered with samples of the western lineage by *rps3-rps19* and *ndhA* but clustered with the samples of the eastern lineage by *chlB-trnQ*, *petD-rpoA*, and *psaC-ndhG*. The concatenated dataset of the five new loci also supported a closer relationship between the Hainan sample and the western lineage and revealed a higher support value compared with each single locus phylogeny (**Figure S2P**).

**Table 3:** Characteristics of newly designed and traditional DNA barcodes of Chinese *Cibotium* plants.

DNA barcode	Product size (bp)	No. of variable sites	No. of parsimony informative sites	PCR primers for new barcodes (5’-3’)
*chlB-trnQ*	714	7 (0.98%)	4 (0.56%)	1f: TCTTTCCCTTTCCGACGTGG 1r: CGGTGACATTTGTTGATCGGT
*petD-rpoA*	799	4 (0.50%)	4 (0.50%)	2f: GCTTGGCCCAATGACCTTT 2r: GTTTCGAAAGCTTTATGGGAACG
*rps3-rps19*	692	5 (0.72%)	5 (0.72%)	3f: TCTTCCATCTGTGCGAACCG 3r: CAACGGACGGGAGCATCTAC
*psaC-ndhG*	800	6 (0.75%)	6 (0.75%)	4f: ACTGAATGTGCCATTGAGTCT 4r: GGTCTGTTTCGTCATCTCGG
*ndhA*	729	3 (0.41%)	3 (0.41%)	5f: TGGGCAAAGTCCGTCTTGTC 5r: CGGAGATGTATGGTAAGCTTCAGA
*chlB-trnQ +petD-rpoA + rps3-rps19 +psaC-ndhG +ndhA*	3734	25 (0.67%)	22 (0.59%)	
*atpA*	1506	1 (0.07%)	1 (0.07%)	
*atpB*	1355	3 (0.22%)	1 (0.07%)	
*rbcL*	1136	3 (0.26%)	0 (0.00%)	
*rps4*	554	0 (0.00%)	0 (0.00%)	
*rbcL-accD*	1445	4 (0.28%)	1 (0.07%)	
*rbcL-atpB*	631	1 (0.16%)	0 (0.00%)	
*trnG-trnR*	945	5 (0.53%)	2 (0.21%)	
*trnL-trnF*	947	5 (0.53%)	4 (0.42%)	
*rps4-trnS*	440	1 (0.23%)	1 (0.23%)	
*rbcL-accD* +*trnG-trnR* +*trnL-trnF* +*rps4-trnS*	3468	15 (0.43%)	8 (0.23%)	

## Discussion

Our study newly assembled plastomes of 25 *Cibotium* samples from China and adjacent regions and presented the conserved structure and composition of which among and within species. A well-supported phylogeny was constructed based on the plastome dataset and revealed three monophyletic clades corresponding to three species, i.e., *C. barometz*, *C. cumingii*, and *C. sino-burmaense*, a new species from NW Yunnan and NE Myanmar. The phylogeny also showed two diverging lineages within *C. barometz* with distinct geographical distribution regions. Additionally, we suggested several DNA barcodes that could accurately identify all these Chinese species and lineages and may benefit conservation and medicinal quality evaluation in practice.

### A new *Cibotium* species from Yunnan–Myanmar

Based on our plastome-based phylogenetic relationship, *C. cumingii* is the one that diverged first among the Chinese *Cibotium* species. The result further distinguished two well-supported sister clades from the remaining samples, one comprising samples distributed in S. China and the Ryukyu Islands corresponding to traditionally recognized *C. barometz*, and the other comprising five samples from NW Yunnan and NE Myanmar (Clade A, **Figure 5**). Plastome characteristics, including IR size and GC content, also support the genetic difference between these Yunnan–Myanmar *Cibotium* and the widespread *C. barometz*. Therefore, we named these Yunnan–Myanmar samples a new species, *Cibotium sino-burmaense*, hereafter.

We compared specimens of *C. sino-burmaense* with *C. barometz* and found obvious differences in pinnules and sori characters (see details in the taxonomic treatment part). We checked the spores of *C. sino-burmaense* and found they shared similar perine features with the two known Chinese species photographed by Gastony (1982), with strongly developed equatorial and distal ridges. However, the equatorial diameter of exospores is significantly larger (41–55 μm) than that of *C. barometz* from S. China (30–45 μm). Because larger spore size is an indicator of higher ploidy levels in some fern taxa (Barrington et al., 1986; Henry et al., 2014), we also compared the genome size of the new species with that of *C. barometz*. The nuclear DNA content of *C. sino-burmaense* was estimated at 4.79 pg/C by flow cytometry with *Capsicum annuum* var. *annuum* (3.38 pg/C, Moscone et al., 2003) as the internal standard (**Figure S3**). This result is very close to the record of *C. barometz* (4.58 pg/C, diploid, Clark et al., 2016), showing no significant ploidy variation signal between the two species.

Three known populations of *C. sino-burmaense* are all endemic to the border of SW China and NE Myanmar. Narrow distribution might be a signal of low genetic diversity and population size reduction (bottleneck), which may lead to inbreeding, affect adaptive potential, facilitate the accumulation of deleterious mutations, and finally hinder the long-term survival of species (Ellegren and Galtier, 2016). Considering the limited sample size of this study, more comprehensive field exploration of adjacent regions is needed to illuminate the accurate habitat range, population size, and anthropogenic influences of this new species. Our newly designed DNA barcode, together with phenotypic differences, could be applied to the research by accurately distinguishing *C. sino-burmaense* and *C. barometz* (**Figures S2K–P**). In addition, genetic information from the nuclear genome should also be utilized to further evaluate the genetic diversity, effective population size, demographic history, and probable conservation necessity of this endemic *Cibotium* species.

### Evolutionary divergence within *C. barometz* and implication to conservation

In recent decades, integrating the principles and methodologies of disciplines such as taxonomy, phylogeny, and evolutionary ecology has become a powerful approach to aiding medicinal discovery, identification, and conservation (Sun et al., 2021; Xu et al., 2021; Zaman et al., 2021). Here, by means of phylogenomic analyses, we clarified the species boundary of the highly demanded medicinal plant *C. barometz*, as our findings supported the phylogenetic difference between it and *C. cumingii* and revealed a new species, *C. sino-burmaense*, distributed at the west edge of its geographical range. Moreover, the plastome-based phylogeny also presented two subclades within *C. barometz* occupying different areas in China (Subclade E and Subclade W, **Figure 5**). Due to the lack of other significant divergence in plastome constitution and morphological features between the two subclades, we considered this finding to be an intraspecific lineage divergence with a geographical pattern. Comparing with previous studies constrained by limited sampling areas (e.g., Wu et al., 2007; You and Deng, 2012), the results of this study revealed the east–west divergence throughout the whole distribution region in S. China. The geographical boundary is close to two general phylogeographic breaks in the Sino-Japanese floristic region, i.e., ca. 105°E and the boundary between the Second and Third ladders of landform in China, as reviewed by Ye et al. (2017). The east and west sides of 105°E are dominated by Pacific and Indian monsoons, respectively (Qiu et al., 2011), while altitude is significantly varied between and within different ladders (Li et al., 2013), which also shapes diverse ecological conditions (Fang et al., 2004). Heterogeneous climate and landform as well as refugia isolation resulted from intensity changes of monsoons may have contributed to the east–west genetic split of *C. barometz*, as demonstrated in other plant lineages (e.g., Bai et al., 2014; Sun et al., 2014; Kou et al., 2016). Additionally, the genetic difference also suggested that the east and west lineages should be considered as at least two management units with respective genetic characters and geographical distributions for conservation (Palsbøll et al., 2007). Due to the limited sampling size and uniparentally inherited feature of the plastome, our study could neither investigate further within-population diversity nor reveal probable hybridization or introgression among linages. In the future, studies with a larger population sampling size of *C. barometz* and biparentally inherited nuclear genome data would evaluate population diversity on a finer scale, detect genetic communications among lineages, trace demographic history backwards, and predict the vulnerability of different lineages under the influence of habitat fragmentation and changing climate.

Germplasm resources play a fundamental role in high-quality genuine medicine production (Ma and Xiao, 1998; Huang et al., 2008; Yao et al., 2020; Cheng et al., 2021; Zhang and Jiang, 2021; Meng et al., 2023; Xu et al., 2023). At present, all the cibotii rhizome slices sold on the market come from natural sources without domestication, which seriously affects medicinal quality (Ju et al., 2012; Yang et al., 2015). Environmental conditions affect the synthesis and accumulation of secondary metabolites, which are usually medicinal components in plants (Li et al., 2020). Therefore, it is expected that *C. barometz* populations growing in habitats with diverse climates and ecology may also exhibit pharmacodynamic differences. The genetic divergence observed in this study will be beneficial for selecting specific high-quality germplasm resources from natural populations for cultivation and for elucidating the influence of multiple external factors on the synthesis pathways of metabolites. Combined with the efficacy information of plants from different locations, the newly designed barcode regions could be applied to the identification of geographical origin, which will probably become a method of medicinal quality evaluation in the future.

### Key to three *Cibotium* species of China

1a. Pinnules on basiscopic side of lower pinnae present or only one absent, rarely with two absent; sori 1–10 per pinnule segments........................................................................................2
2a. Pinnules on acroscopic and basisicopic sides of a pinna nearly equal in length; apex of pinnule segments apiculate; sori oblong, usually 1–5 pairs per pinnule segment; average exospore equatorial diameter less than 43 μm...1. *C. barometz*
2b. Pinnules on basiscopic side of a pinna much shorter (c. 1/2) than those on the acroscopic side; apex of pinnule segments acute; sori oblong to spherical, usually 4–8 and sometimes over 10 pairs per pinnule segment; average exospore equatorial diameter more than 45 μm...............................................................2. *C. sino-burmaense*
1b. Pinnules on basiscopic side of lower pinnae usually three lacking; sori usually one or two per pinnule segment...................................................................3. *C. cumingii*

### Taxonomic treatment

(1) *Cibotium barometz* (L.) J. Sm., London J. Bot. 1 (1842) 437.

≡ *Polypodium barometz* L., Sp. Pl. 2 (1753) 1092.

≡ *Aspidium barometz* (L.) Willd., Sp. Pl., ed. 4 [Willdenow] 5 (1810) 268.

≡ *Nephrodium barometz* (L.) Sweet, Hort. Brit. [Sweet], ed. 2. (1830) 580.

≡ *Dicksonia barometz* (L.) Link, Fil. Spec. (1841) 166.

Neotype (designated by Mazumdar in Nordic J. Bot. 34(4): 465. 2016): —China. Guangxi, SE of Shang-sze (Shangsi) District, Shap Man Taai Shan (Shiwandashan), near Hoh Lung village, 16 Jun 1933, *W.T. Tsang 22473* (S No. S14-33459 [image!])

= *Balantium glaucescens* Link, Fil. Spec. (1841) 40.

Type: —Not designated.

= *Cibotium glaucescens* Kunze, Farnkräuter 1 (1841) 63, t.31.

Type: —Not designated.

*= Cibotium assamicum* Hook., Sp. Fil. [W. J. Hooker] 1 (1844) 83, t.29B.

Holotype: —India. Assam, *Mrs. Mack s.n.* (not traced).

= *Dicksonia assamicum* Griff., Notul. 2 (1849) 607.

Lectotype (designated here): —India. Assam, *Griffith s.n.* (K barcode K001090393 [image!]).

= *Cibotium djambianum* Hassk., Fil. Jav. 1 (1856) 61.

Type: —Not designated.

Distribution: —China (Chongqing, Fujian, Guangdong, Guangxi, Guizhou, Hainan, Hunan, Jiangxi, Sichuan, Taiwan, Xizang, Yunnan, Zhejiang), Japan (Ryukyu Islands), Indonesia (Java to Sumatra), Malaysia, Myanmar, Thailand, Vietnam.

**Figure 7:**
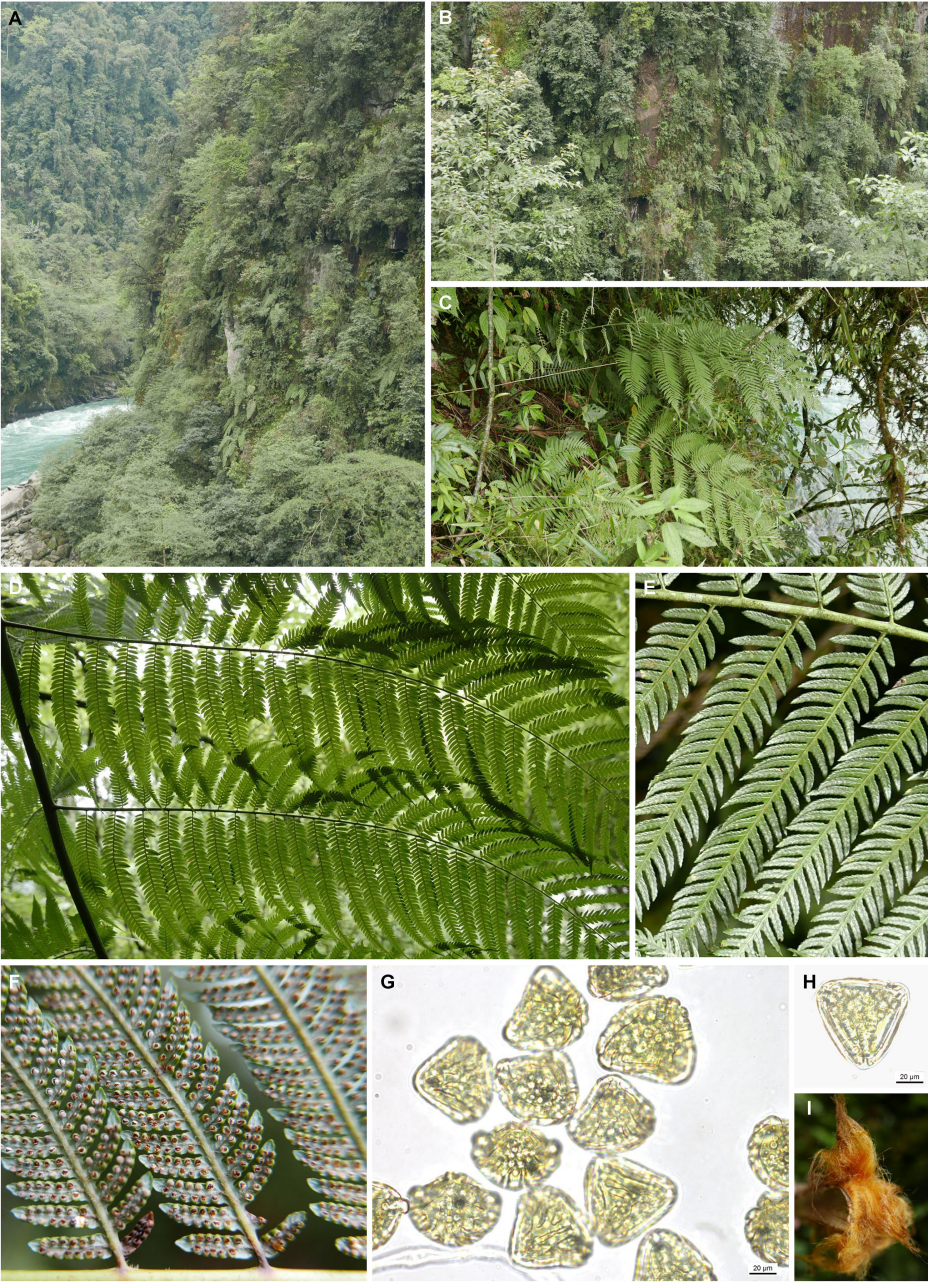
Habitat and morphology of *Cibotium sino-burmaense* sp. nov. from Dulongjiang, Gongshan, Yunnan, China. **(A, B)** Habitat. **(C)** Habit. **(D)** Pinnae on the abaxial side. **(E)** Pinnules on the adaxial surface. **(F)** Pinnules with opened sori. **(G, H)** Spores under light microscopy. **(I)** Golden filiform hairs on the stipe base. Photographs by X.-C. Zhang **(A–F, I)**, and S.-Q. Liang **(G, H)**.

(2) *Cibotium sino-burmaense* X.C. Zhang & S.Q. Liang, sp. nov. (**Figure 7**)

Diagnosis: —This new species resembles *C. barometz* and *C. cumingii*, differing from the former in the significantly shortened pinnule length on basiscopic side, as well as acute apex and more sori of pinnule segments, and from the latter in the denser sori per pinnule segment and presence of the second and third pinnules on the basiscopic side of lower pinnae.

Holotype: —China. Yunnan: Gongshan county, Dulongjiang Township, 2 May 2022, *X.C. Zhang 12880* (PE!).

Note: —The holotype consists of a single large frond mounted on fifteen herbarium sheets, labeled “sheet 1” to “sheet 15”.

Description: —Rhizome prostrate, stout, densely covered with shiny yellowish brown long hairs. Stipes thick, up to 80 cm or more, dark brown to purplish black at base and becoming green upwards, covered with long hairs similar to those on rhizome at base, upper part covered with small, appressed flaccid hairs. Lamina ovate, 2-pinnate-pinnatifid, up to 3 m, subleathery, adaxial surface deep green, abaxial surface glaucous, with small flaccid hairs on midrib; pinna 8–10 pairs, alternate, stalked, medial pinnae 60–80 × 20–30 cm, basal pinna pairs reduced slightly; pinnules more than 30 pairs per lower pinna, shortly stalked, up to 20 cm on the acroscopic side, 10-14 cm on the basiscopic side; pinnule segments, alternate, slightly falcate, with acute apex, margins crenulate to serrulate-serrate. Sori oblong to spherical, usually 4–8 and sometime over 10 pairs at base of lower pairs of pinnule segments; indusia bivalvate, outer indusia larger, orbicular, inner significantly smaller, oblong. Spores pale yellowish, with strongly developed equatorial and distal ridges.

Etymology: —*Sino-burmaense* is derived from the known distribution of this species along China–Myanmar border.

Additional Specimens Examined: —China. Yunnan: Gongshan county, Dulongjiang Township, 23 Jan 2017, *X.C. Zhang & al. 8134*; Fugong County, 26 April 2022, *X.C. Zhang 12831.* Myanmar. Kachin: Htawgaw, April 1925, *G. Forrest 26496* (PE barcode 01654827, 01654828, 00388348).

Distribution and habitat: —China (NW Yunnan), Myanmar (Kachin). On cliff with open canopy.

(3) *Cibotium cumingii* Kunze, Farrnkräuter 1 (1841) 64, 65.

≡ *Cibotium barometz* var. *cumingii* (Kunze) C. Chr., Index Filic. 3 (1905) 183.

Lectotype (designated here): —Philippines. Luzon, *H. Cuming 123* (K barcode K000376224 [image!]; isolectotypes: K barcode K000376225 [image!], K000376228, K000376229, K000376231, K000376232; BM barcode BM001048122 [image!]; E barcode E00822366 [image!], E00822367 [image!], E00822369 [image!], E00822373 [image!]; P barcode P00633260 [image!], P00633261 [image!], P00633262 [image!]; US barcode 00134826 [image!]; Z barcode Z-000002072 [image!]).

= *Cibotium crassinerve* Rosenst., Meded. Rijks-Herb. 31 (1917) 4.

Lectotype (designated here): —Philippines. Luzon, Benguet, Dec 1908, *H. M. Curran & M. L. Merritt 15800* (L barcode L 0051165 [image!]; isolectotype: MICH No. 1190172 [image!]).

= *Cibotium taiwanense* C. M. Kuo, Taiwania 30 (1985) 56, 57.

Lectotype (designated here): —China. Taiwan, Hsinchu, Chu-tong, August 1972, *C. M. Kuo 1703* (TAI No. 149443 [image!]; isolectotypes: TAI No. 148725 [image!], 150173 [image!]).

Distribution: —China (Taiwan), Japan (Ryukyu Islands), Philippines.

## Conclusion

This study presents the conserved structure and gene composition of the chloroplast genome within *Cibotium* from China. Based on phylogenomic analyses, we constructed a well-supported phylogeny of Chinese *Cibotium* and indicated that there are three species distributed in China, namely *C. barometz*, *C. cumingii*, and *C. sino-burmaense*, an overlooked cryptic species from NW Yunnan and NE Myanmar. Moreover, our results uncovered the east-west lineage divergence in *C. barometz*. We also evaluated the species resolution of nine old cpDNA loci and suggested five new cpDNA barcodes that are capable of identifying all the above-mentioned species and lineages of Chinese *Cibotium* accurately. In conclusion, our findings will improve people’s understanding of the germplasm resource diversity of this endangered medicinal plant group and play a guiding role in its wild population conservation and medical value exploitation.

## Data availability statement

The datasets presented in this study can be found in online repositories. The names of the repository/repositories and accession number(s) can be found below: https://www.ncbi.nlm.nih.gov/genbank/, OQ721080-OQ721104.

## Author contributions

X-CZ, K-XL and R-HJ designed this study. FW, L-MT, BQ, Y-YC and Y-HH collected and cultivated plant materials of this study. S-QL performed experiments, analyzed the data, and wrote the manuscript. All authors contributed to the article and approved the submitted version.
