# Supplementary material for: Phylogenomic analysis, cryptic species discovery, and DNA barcoding of the genus Cibotium in China based on plastome data
Source: Front Plant Sci. 2023 Jun 6;14:1183653. doi: 10.3389/fpls.2023.1183653 (PMC10279961; doi:10.3389/fpls.2023.1183653)
Supplement: Supplementary file 1 [file DataSheet_1.pdf]

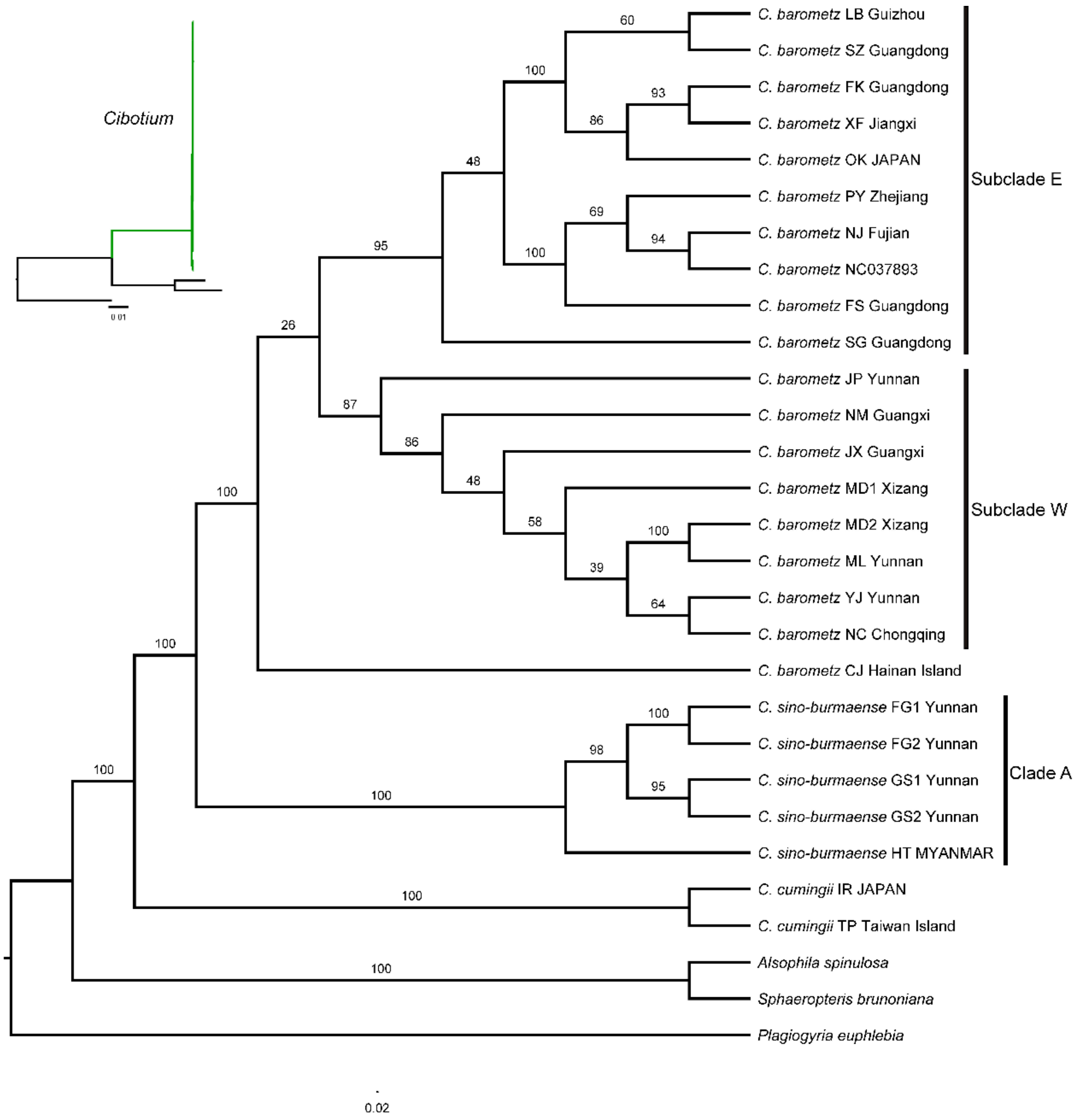

FIGURE S1 Maximum likelihood cladogram of the *Cibotium* plant from China and adjacent regions inferred from whole length plastomes. Numbers above branches are bootstrap values (MLBS). The corresponding phylogram showing branch length is placed at the upper left corner.

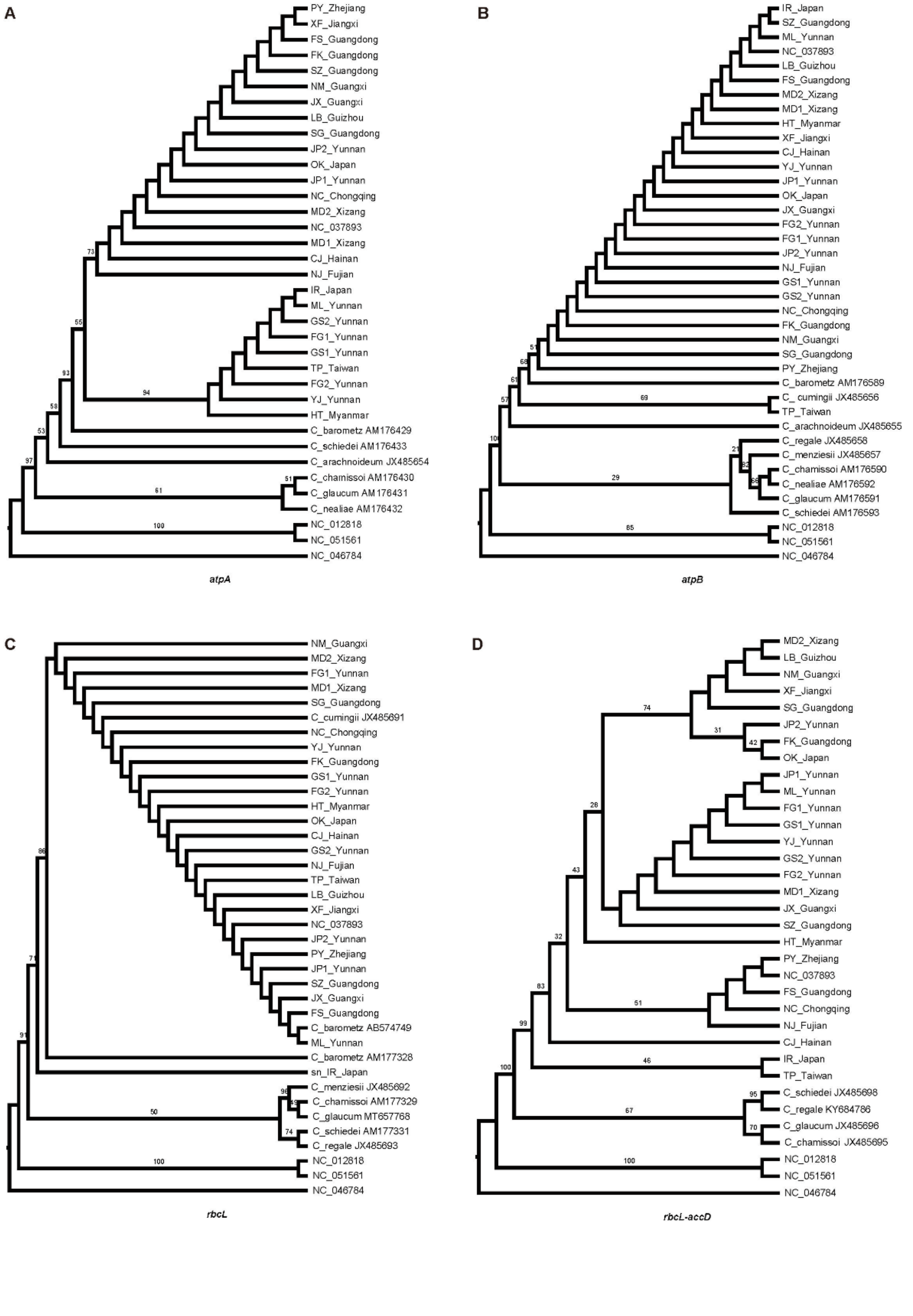

A
PY_Zhejiang
XF_Jiangxi
FS_Guangdong
FK_Guangdong
SZ_Guangdong
NM_Guangxi
JX_Guangxi
LB_Guizhou
SG_Guangdong
JP2_Yunnan
OK_Japan
JP1_Yunnan
NC_Chongqing
MD2_Xizang
NC_037893
MD1_Xizang
CJ_Hainan
NJ_Fujian
IR_Japan
ML_Yunnan
GS2_Yunnan
FG1_Yunnan
GS1_Yunnan
TP_Taiwan
FG2_Yunnan
YJ_Yunnan
HT_Myanmar
C_barometz AM176429
C_schiedei AM176433
C_arachnoideum JX485654
C_chamissoi AM176430
C_glaucum AM176431
C_nealiae AM176432
NC_012818
NC_051561
NC_046784
atpA
B
IR_Japan
SZ_Guangdong
ML_Yunnan
NC_037893
LB_Guizhou
FS_Guangdong
MD2_Xizang
MD1_Xizang
HT_Myanmar
XF_Jiangxi
CJ_Hainan
YJ_Yunnan
JP1_Yunnan
OK_Japan
JX_Guangxi
FG2_Yunnan
FG1_Yunnan
JP2_Yunnan
NJ_Fujian
GS1_Yunnan
GS2_Yunnan
NC_Chongqing
FK_Guangdong
NM_Guangxi
SG_Guangdong
PY_Zhejiang
C_barometz AM176589
C_ cumingii JX485656
TP_Taiwan
C_arachnoideum JX485655
C_regale JX485658
C_menziesii JX485657
C_chamissoi AM176590
C_nealiae AM176592
C_glaucum AM176591
C_schiedei AM176593
NC_012818
NC_051561
NC_046784
atpB
C
NM_Guangxi
MD2_Xizang
FG1_Yunnan
MD1_Xizang
SG_Guangdong
C_cumingii JX485691
NC_Chongqing
YJ_Yunnan
FK_Guangdong
GS1_Yunnan
FG2_Yunnan
HT_Myanmar
OK_Japan
CJ_Hainan
GS2_Yunnan
NJ_Fujian
TP_Taiwan
LB_Guizhou
XF_Jiangxi
NC_037893
JP2_Yunnan
PY_Zhejiang
JP1_Yunnan
SZ_Guangdong
JX_Guangxi
FS_Guangdong
C_barometz AB574749
ML_Yunnan
C_barometz AM177328
sn_IR_Japan
C_menziesii JX485692
C_chamissoi AM177329
C_glaucum MT657768
C_schiedei AM177331
C_regale JX485693
NC_012818
NC_051561
NC_046784
rbcL
D
MD2_Xizang
LB_Guizhou
NM_Guangxi
XF_Jiangxi
SG_Guangdong
JP2_Yunnan
FK_Guangdong
OK_Japan
JP1_Yunnan
ML_Yunnan
FG1_Yunnan
GS1_Yunnan
YJ_Yunnan
GS2_Yunnan
FG2_Yunnan
MD1_Xizang
JX_Guangxi
SZ_Guangdong
HT_Myanmar
PY_Zhejiang
NC_037893
FS_Guangdong
NC_Chongqing
NJ_Fujian
CJ_Hainan
IR_Japan
TP_Taiwan
C_schiedei JX485698
C_regale KY684786
C_glaucum JX485696
C_chamissoi JX485695
NC_012818
NC_051561
NC_046784
rbcL-accD

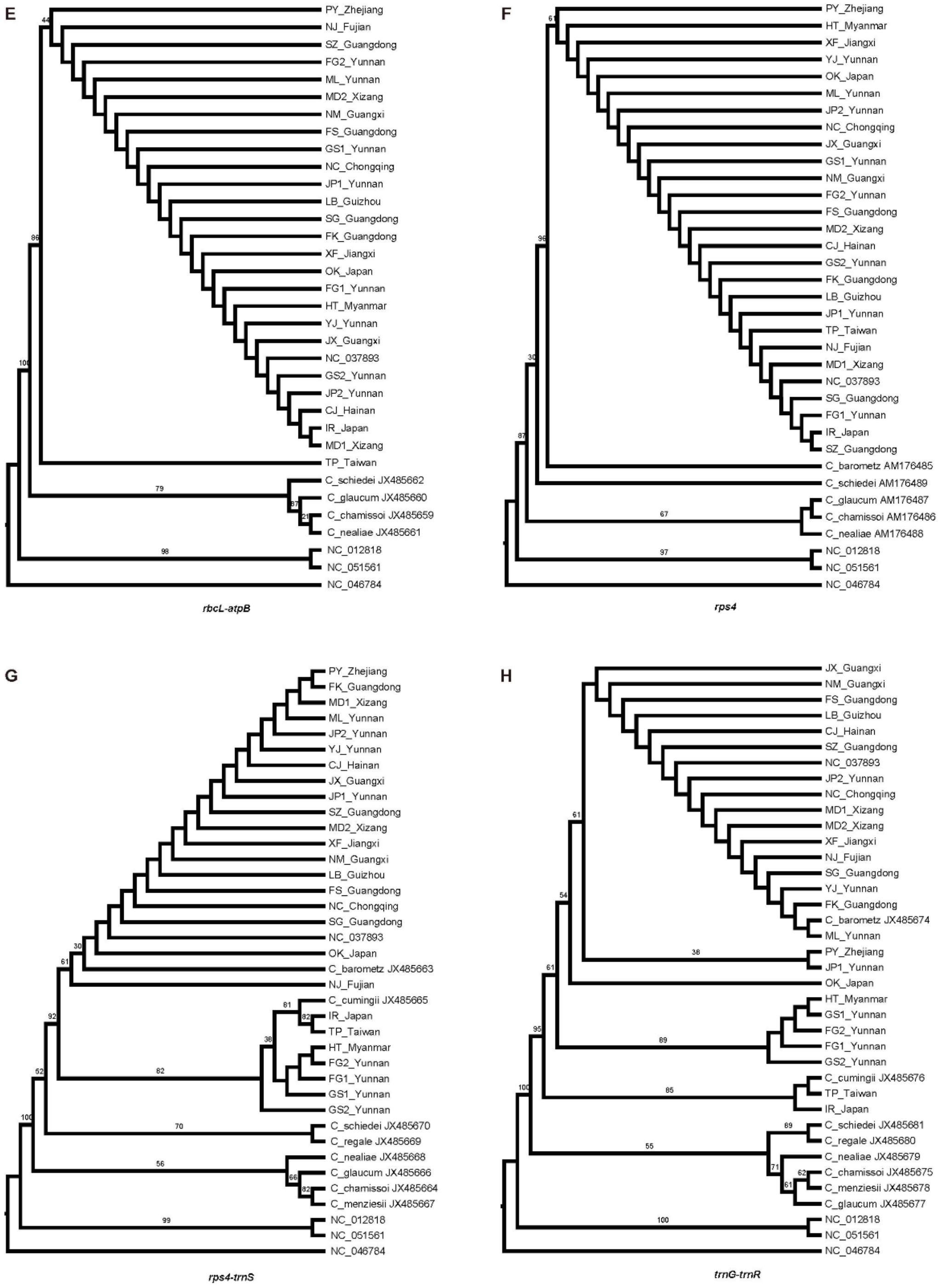
E
PY_Zhejiang
NJ_Fujian
SZ_Guangdong
FG2_Yunnan
ML_Yunnan
MD2_Xizang
NM_Guangxi
FS_Guangdong
GS1_Yunnan
NC_Chongqing
JP1_Yunnan
LB_Guizhou
SG_Guangdong
FK_Guangdong
XF_Jiangxi
OK_Japan
FG1_Yunnan
HT_Myanmar
YJ_Yunnan
JX_Guangxi
NC_037893
GS2_Yunnan
JP2_Yunnan
CJ_Hainan
IR_Japan
MD1_Xizang
TP_Taiwan
C_schiedei JX485662
C_glaucum JX485660
C_chamissoi JX485659
C_nealiae JX485661
NC_012818
NC_051561
NC_046784
rbcL-atpB
F
PY_Zhejiang
HT_Myanmar
XF_Jiangxi
YJ_Yunnan
OK_Japan
ML_Yunnan
JP2_Yunnan
NC_Chongqing
JX_Guangxi
GS1_Yunnan
NM_Guangxi
FG2_Yunnan
FS_Guangdong
MD2_Xizang
CJ_Hainan
GS2_Yunnan
FK_Guangdong
LB_Guizhou
JP1_Yunnan
TP_Taiwan
NJ_Fujian
MD1_Xizang
NC_037893
SG_Guangdong
FG1_Yunnan
IR_Japan
SZ_Guangdong
C_barometz AM176485
C_schiedei AM176489
C_glaucum AM176487
C_chamissoi AM176486
C_nealiae AM176488
NC_012818
NC_051561
NC_046784
rps4
G
PY_Zhejiang
FK_Guangdong
MD1_Xizang
ML_Yunnan
JP2_Yunnan
YJ_Yunnan
CJ_Hainan
JX_Guangxi
JP1_Yunnan
SZ_Guangdong
MD2_Xizang
XF_Jiangxi
NM_Guangxi
LB_Guizhou
FS_Guangdong
NC_Chongqing
SG_Guangdong
NC_037893
OK_Japan
C_barometz JX485663
NJ_Fujian
C_cumingii JX485665
IR_Japan
TP_Taiwan
HT_Myanmar
FG2_Yunnan
FG1_Yunnan
GS1_Yunnan
GS2_Yunnan
C_schiedei JX485670
C_regale JX485669
C_nealiae JX485668
C_glaucum JX485666
C_chamissoi JX485664
C_menziesii JX485667
NC_012818
NC_051561
NC_046784
rps4-trnS
H
JX_Guangxi
NM_Guangxi
FS_Guangdong
LB_Guizhou
CJ_Hainan
SZ_Guangdong
NC_037893
JP2_Yunnan
NC_Chongqing
MD1_Xizang
MD2_Xizang
XF_Jiangxi
NJ_Fujian
SG_Guangdong
YJ_Yunnan
FK_Guangdong
C_barometz JX485674
ML_Yunnan
PY_Zhejiang
JP1_Yunnan
OK_Japan
HT_Myanmar
GS1_Yunnan
FG2_Yunnan
FG1_Yunnan
GS2_Yunnan
C_cumingii JX485676
TP_Taiwan
IR_Japan
C_schiedei JX485681
C_regale JX485680
C_nealiae JX485679
C_chamissoi JX485675
C_menziesii JX485678
C_glaucum JX485677
NC_012818
NC_051561
NC_046784
trnG-trnR

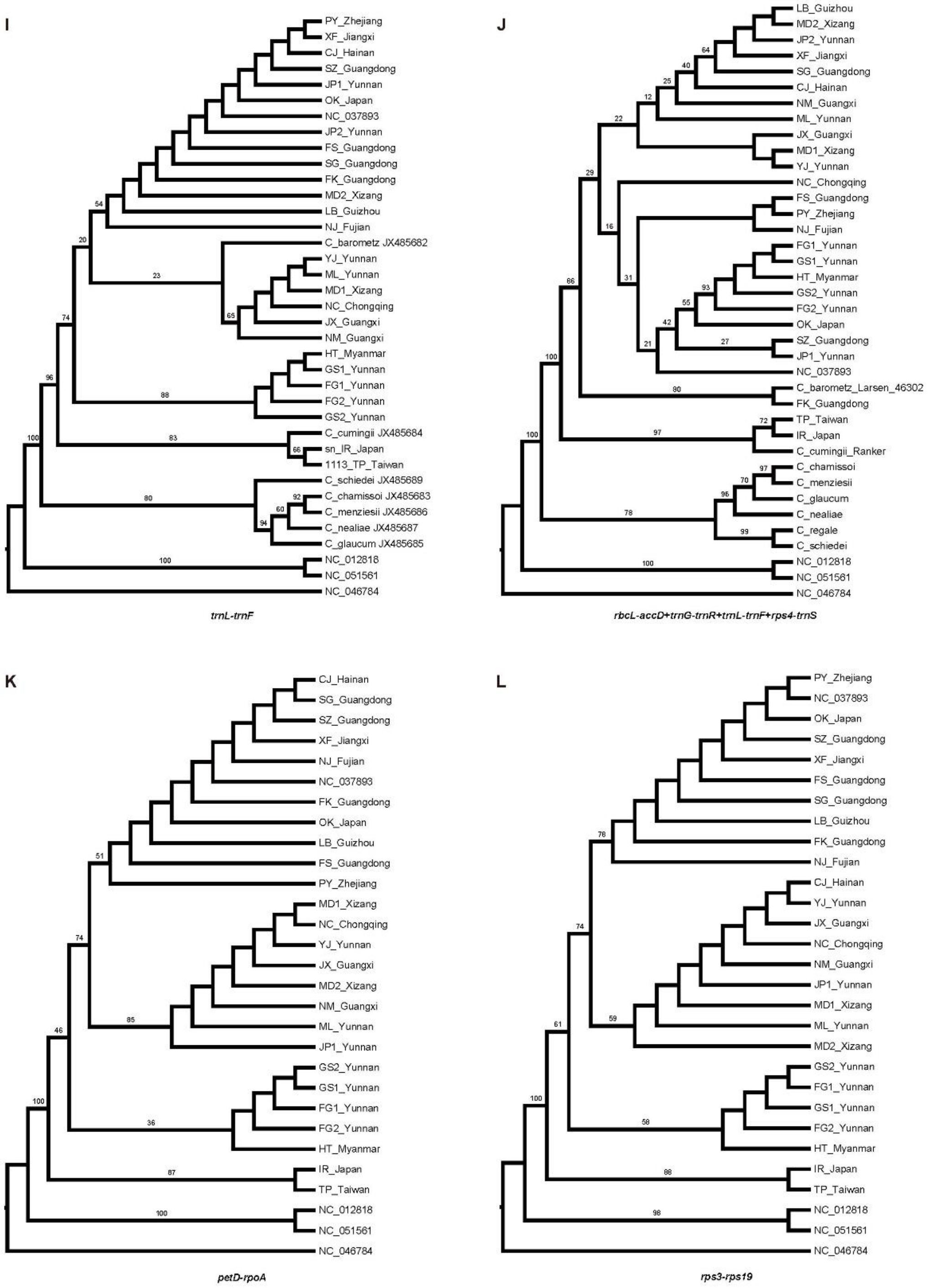
I
PY_Zhejiang
XF_Jiangxi
CJ_Hainan
SZ_Guangdong
JP1_Yunnan
OK_Japan
NC_037893
JP2_Yunnan
FS_Guangdong
SG_Guangdong
FK_Guangdong
MD2_Xizang
LB_Guizhou
NJ_Fujian
C_barometz JX485682
YJ_Yunnan
ML_Yunnan
MD1_Xizang
NC_Chongqing
JX_Guangxi
NM_Guangxi
HT_Myanmar
GS1_Yunnan
FG1_Yunnan
FG2_Yunnan
GS2_Yunnan
C_cumingii JX485684
sn_IR_Japan
1113_TP_Taiwan
C_schiedei JX485689
C_chamissoi JX485683
C_menziesii JX485686
C_nealiae JX485687
C_glaucum JX485685
NC_012818
NC_051561
NC_046784
trnL-trnF
J
LB_Guizhou
MD2_Xizang
JP2_Yunnan
XF_Jiangxi
SG_Guangdong
CJ_Hainan
NM_Guangxi
ML_Yunnan
JX_Guangxi
MD1_Xizang
YJ_Yunnan
NC_Chongqing
FS_Guangdong
PY_Zhejiang
NJ_Fujian
FG1_Yunnan
GS1_Yunnan
HT_Myanmar
GS2_Yunnan
FG2_Yunnan
OK_Japan
SZ_Guangdong
JP1_Yunnan
NC_037893
C_barometz_Larsen_46302
FK_Guangdong
TP_Taiwan
IR_Japan
C_cumingii_Ranker
C_chamissoi
C_menziesii
C_glaucum
C_nealiae
C_regale
C_schiedei
NC_012818
NC_051561
NC_046784
rbcL-accD+trnG-trnR+trnL-trnF+rps4-trnS
K
CJ_Hainan
SG_Guangdong
SZ_Guangdong
XF_Jiangxi
NJ_Fujian
NC_037893
FK_Guangdong
OK_Japan
LB_Guizhou
FS_Guangdong
PY_Zhejiang
MD1_Xizang
NC_Chongqing
YJ_Yunnan
JX_Guangxi
MD2_Xizang
NM_Guangxi
ML_Yunnan
JP1_Yunnan
GS2_Yunnan
GS1_Yunnan
FG1_Yunnan
FG2_Yunnan
HT_Myanmar
IR_Japan
TP_Taiwan
NC_012818
NC_051561
NC_046784
petD-rpoA
L
PY_Zhejiang
NC_037893
OK_Japan
SZ_Guangdong
XF_Jiangxi
FS_Guangdong
SG_Guangdong
LB_Guizhou
FK_Guangdong
NJ_Fujian
CJ_Hainan
YJ_Yunnan
JX_Guangxi
NC_Chongqing
NM_Guangxi
JP1_Yunnan
MD1_Xizang
ML_Yunnan
MD2_Xizang
GS2_Yunnan
FG1_Yunnan
GS1_Yunnan
FG2_Yunnan
HT_Myanmar
IR_Japan
TP_Taiwan
NC_012818
NC_051561
NC_046784
rps3-rps19

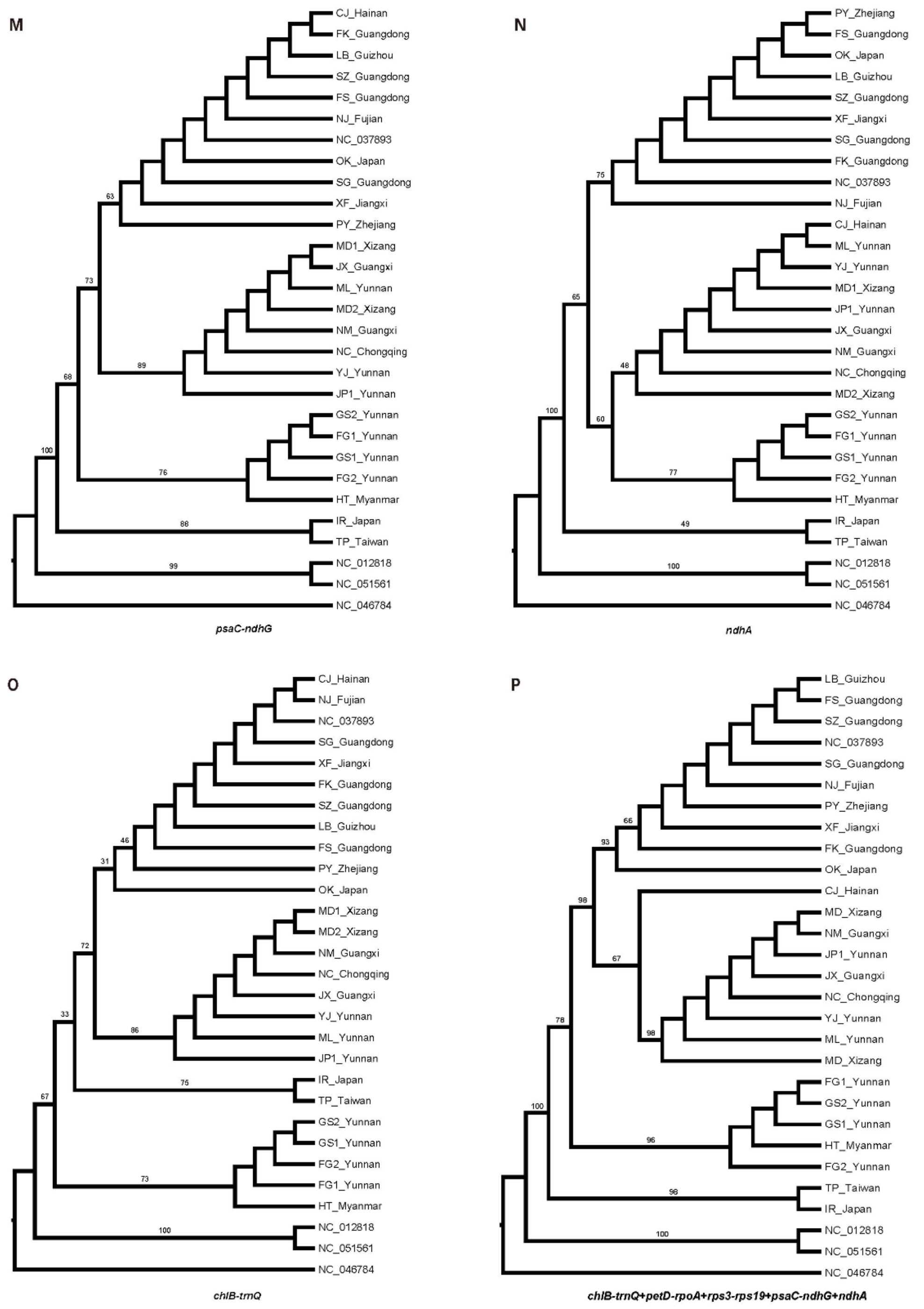

FIGURE S2 Maximum likelihood cladogram of the *Cibotium* built with different cpDNA barcode loci. Numbers above branches are bootstrap values.

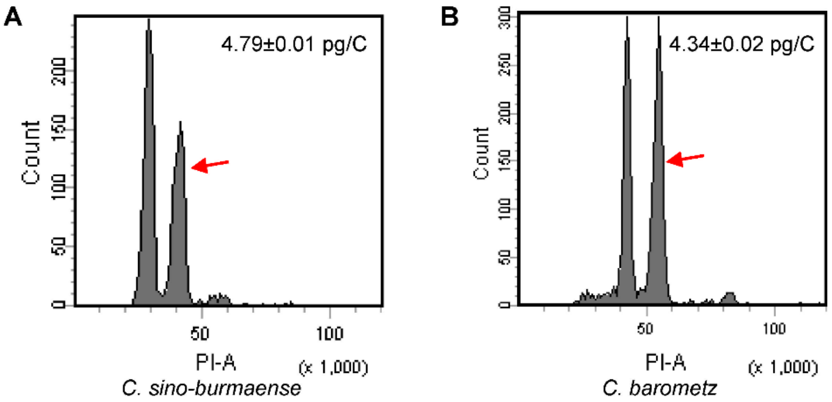

FIGURE S3 Histograms of fluorescence intensity in two *Cibotium* species. The peak marked with a red arrow represents *Cibotium* sample, while the other peak represents the internal standard *Capsicum annuum* var. *annuum* (nuclear DNA content: 3.38 pg/C).
